# Richness estimation in microbiome data obtained from denoising pipelines

**DOI:** 10.1016/j.csbj.2021.12.036

**Published:** 2022-01-10

**Authors:** Sven Kleine Bardenhorst, Marius Vital, André Karch, Nicole Rübsamen

**Affiliations:** a Institute of Epidemiology and Social Medicine, University of Münster, Münster, Germany; b Institute of Medical Microbiology and Hospital Hygiene, Hannover Medical School, Hannover, Germany

**Keywords:** Microbiome, Rarefaction, Denoising, Sequencing depth, Sub-sampling

## Abstract

The quantification of richness within a sample—either measured as the number of observed species or approximated by estimation—is a common first step in microbiome studies and is known to be highly dependent on sequencing depth, which itself is highly variable between samples. Rarefaction curves serve as a tool to investigate this dependency and it is often argued that after rarefying data—sub-sampling to an equal sequencing depth—richness estimates no longer depend on sequencing depth. However, the estimation of richness from data obtained by high throughput sequencing methods and processed by current bioinformatics pipelines may be subject to various sources of variation related to sequencing depth. Those that may confound inference based on richness estimates and cannot be solved by sub-sampling. We investigated how pipeline settings in DADA2 and deblur affect estimates of richness and showed that the use of rarefaction and sub-sampling is inappropriate when default pipeline settings are applied. Furthermore, we showed how independent sample-wise processing established spurious correlations between sequencing depth and richness estimations in data produced by DADA2 and how this problem can be solved by a pooled processing approach.

## Graphical abstract

**Figure:**
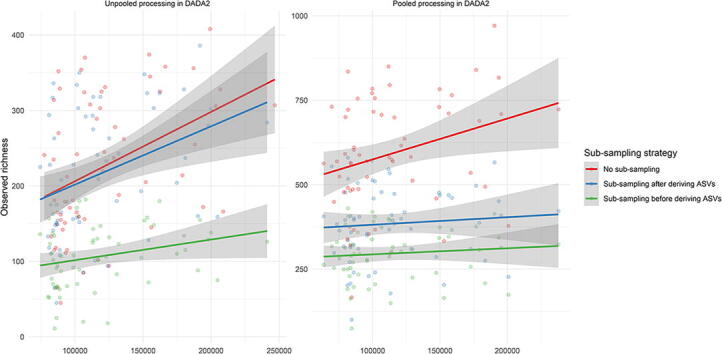

## 1 Introduction

The estimation of alpha diversity—the diversity within a given environment—is a common first step when investigating ecological communities. The most common and simple measure of alpha diversity is the richness in terms of either the observed or estimated number of species within a given community. In current microbiome research, however, communities are not directly observed, but inferred from biological samples using high-throughput sequencing methods. These methods make use of the bacterial gene content in a biological sample to infer its microbial community structure. However, the number of reads (i.e. the number of gene sequences) that are sequenced per sample can be highly variable for purely technical reasons. As a higher overall read count (sequencing depth) increases the chance of detecting rare sequences, richness is positively correlated with sequencing depth.

**Fig. 1:**
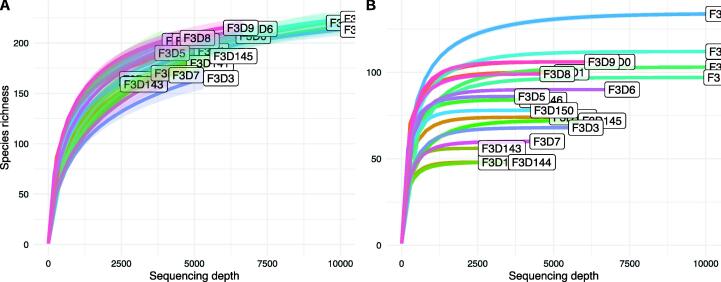
Two common patterns in rarefaction curves. Panel **A** shows a smooth incline in observed richness. Panel **B** shows a steep incline and early plateau for all samples.

Rarefaction is a common yet strongly criticized method developed to assess the coverage of detected sequences in a sample by plotting the number of observed sequences (or taxa, i.e. genera or species) as a function of sequencing depth. When this rarefaction curve reaches a plateau (Fig. 5 **B**), only few new sequences are detected with increasing sequencing depth. Therefore, one may conclude that the sample sufficiently covered the original community it was taken from. Rarefaction is often used with the intention to assess whether a fair comparison of richness between microbial communities measured with unequal sequencing depths is possible. Another strategy typically applied is to sub-sample all communities to an equal sequencing depth – usually the lowest observed sequencing depth among all samples – to reduce the influence of sequencing depth inequalities on richness estimations (See Fig. 1).

In recent microbiome research, rarefaction curves as well as sub-sampling are still frequently used. However, current pipelines (i.e. DADA2 [1] or deblur [2]) use a fundamentally different approach as compared to former bioinformatics pipelines by incorporating an error model to correct for sequencing errors (denoising) and derive exact sequence variants, instead of grouping similar sequences into operational taxonomic units (OTU) to account for sequencing errors. Although this approach allows for computational efficient sample-wise processing, it is not clear how these differences affect sub-sampling and rarefaction in data obtained by those denoising pipelines.

The aim of this study is to investigate the effects of rarefaction and sub-sampling on alpha diversity estimated from data processed using denoising pipelines based on the example of DADA2 [1] and deblur [2] and to assess which aspects of the pipeline affect richness estimations.

## 2 Methods

To assess how different processing approaches affect rarefaction and sub-sampling followed by estimation of alpha diversity, several datasets were processed using DADA2 and deblur with different pipeline setups. DADA2 and deblur are pipelines that aim to infer sample sequences exactly up to single nucleotide differences. The resulting error corrected sequences are referred to as amplicon sequence variants (ASV).

All datasets were published previously and are publicly available via the European Nucleotide Archive. Derivation of ASVs in all datasets was achieved using four different approaches in DADA2 (v.1.20.0). At first, among all approaches, the following DADA2-parameters remained fixed: maxEE = 2, truncLen = (250,250), trimLeft = c(17,21), rm.phix = TRUE, while all other parameters were pipeline default settings. In addition, chimeric reads were filtered using the *removeBimeraDenovo*-function. For further explanation of these parameters, please refer to [1].

For DADA2, the difference in the four processing approaches is the combination of two argumentsin the DADA2 pipeline. The first argumentdefines whether to use standard sample-wise processing (**1**) or to pool information across samples (**2**). DADA2, by default, processes samples independently (sample-wise). This is computationally efficient, but may result in poor sensitivity to very low abundant sequences. For example, suppose we have two samples and the same sequence was observed once in the first sample and five times in the second. Due to independent sample-wise processing, the sequence will be discarded in the first sample, but not in the second sample. However, pooling information would keep those reads in all samples and increase sensitivity. The second argument is the whether to process data without prior information (**a**) or to include prior information about sequences (**b**). Regardless of whether we process data pooled or unpooled, we can pass a list of sequences that we expect to find as an argument to the main DADA2-function *dada*. If a singleton is detected in a sample, it will not be discarded if it is present in the list of prior sequences.

To assess the effects of sub-sampling, two different approaches were used. In the first approach, the sub-sampling was performed before deriving ASVs by randomly sampling raw sequence reads using the *FastqSampler*-function in the ShortRead package(v.1.50.0) (pre-ASV). The second approach performed sub-sampling on the processed data after deriving ASVs (post-ASV) using the *rarefy*_*even*_*depth()*-function of the phyloseq package (v.1.36.0) [3].

The combination of sub-sampling approach and pipeline setup resulted in 3×2×2=12different scenarios per dataset ((Full data/ pre-ASV sub-sampling/ post-ASV sub-sampling) × (pooled/ unpooled) x (prior/ no prior)).

**Fig. 2:**
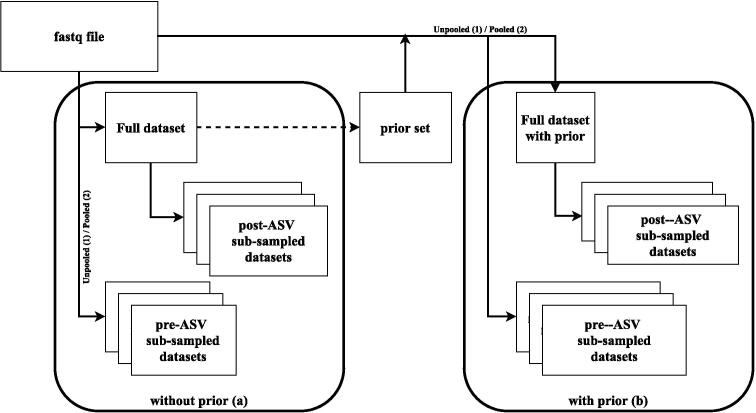
Workflow of pipelines to generate rarefied datasets.

Note, that the resulting sequences of the scenarios without prior and without sub-sampling served as set of prior sequences for all scenarios with prior. A detailed overview of the workflow is shown in Fig. 2.

In addition to DADA2, the analysis were replicated using deblur [4] as implementet in QIIME2 [2]. Deblur is a pipeline that, similar to DADA2, uses a denoising approach to derive amplicon sequence variants with a resolution up to a single nucleotide. Just as DADA2, deblur processes data sample-wise. However, deblur does not allow pooling of information, which prevents direct comparison of DADA2 and deblur. To have comparable setups, two different scenarios were applied for deblur. In the first scenario, the data was processed with the default setting in which sequences with less than one reads will be discarded. In the second scenario, to achieve similar increase in sensitivity to singletons as in DADA2 pooled processing, the minimal read count of a single sequence was set to one, to keep singleton reads in all samples. To achieve maximum comparability, the data that was already filtered and trimmed by the DADA2 pipeline was used as input for deblur. Note, that, although deblur follows a similar approach as DADA2, the two core algorithms are fundamentally different.

For all scenarios, alpha diversity in terms of observed richness, Shannon index, Simpson index, Chao1 index [5], the ACE index [6], Good’s coverage [7], Margalef’s diversity index and Menhinick’s diversity index were estimated at the ASV level using the packages HillR (v.0.5.1 [8]),fossil (v.0.4.0 [9]),abdiv (v.0.2.0 [10]),and QsRutils (v.0.1.5 [11]). Observed richness, Shannon index and Simpson index were calculated through Hill numbers. Hill numbers are generalizations of alpha diversity that converge to observed richness (order zero), Shannon index (order one) and Simpson index(order two), respectively [12]. Rarefaction curves were obtained using the package iNext (v.2.0.20 [13]) and adjusted using the ggplot2-package (v.3.3.5 [14]). Due to technical reasons, some observations may lose a lot of sequences when sub-sampled and will be discarded from the sample. Therefore, we selected only observations with at least 10,000 reads in the full (not sub-sampled) dataset and at least 2000 reads after sub-sampling. Moreover, observations were only kept when they were available among all 12 scenarios. All analyses and processing steps were performed in the R statistical programming language [15] running on an high-performance computing cluster. All R scripts are available at  https://github.com/SvenKB/RichnessEstimationDenoising.

## 3 Results

The analyses showed that alpha diversity estimates as well as correlations with sequencing depth are highly dependent on the pipeline setup that was used to derive ASVs. The use of pooled processing allowed to share information across samples, resulting in a higher sensitivity for low abundant reads and may produce singletons within single samples. The use of prior information resulted in a similar, partial, pooling effect. The sequences detected in one sample will be used as information for other samples. Therefore, a previously detected singleton may now be kept, when the same sequence is in the list of prior sequences (See Table 1).

**Table 1:** Description of data used for example analyses. All datasets were publicly available at the European Nucleotide Archive under the given ENA accession number.

Reference	Body site	Diagnosis	16S rRNA region	Sample size	ENA accession	Sub-sampling depth
Almeida-Santos et al., 2021	Oral (Saliva)	Diabetes/ healthy controls	V3-V4	Cases = 25 HC = 25	PRJNA679485	11150
Manor et al., 2018	Gut (Stool)	No diagnosis	V3-V4	N = 60^1^	PRJNA471742	21092
Aho et al., 2019 ^2^	Gut (Stool)	Parkinson’s disease/ healthy controls	V3-V4	PD = 64 HC = 64	PRJEB27564	12341

^1^ Note, that the original sample size was N = 648. For feasibility, only a subset of the original data was used for our analyses.

^2^ Due to repeated measurements, two samples were available per observation. For our analyses, we selected the more recent sample.

**Table 2:** Formal definition of calculated diversity indices. $p_{i}$ refers to the relative abundance of ASV i. q refers to the order of the hill number. $S_{\mathrm{obs}}$ refers to the number of observed ASVs. $S_{n_{1}}$ and $S_{n_{2}}$ refer to the number of singletons and doubletons, respectively. $S_{\mathrm{abund}}$ and $S_{\mathrm{rare}}$ refers to the number of ASVs with absolute abundance above or below 10, respectively. $N_{\mathrm{rare}}$ refers to the total number of reads in the rare ASVs.

Index	Formula
Observed richness	$S_{\mathrm{obs}}={}^{0}D=\left(\sum_{i=1}^{S}p_{i}^{0}\right)$
Shannon index	$H\prime =-\sum_{i=1}^{S_{\mathrm{obs}}}p_{i}\cdot \ln p_{i}={}^{1}D={\left(\sum_{i=1}^{S_{\mathrm{obs}}}p_{i}^{1}\right)}^{1/(1-(q\to 1))}$
Simpson index	$D_{\mathrm{Sim}}={}^{2}D={\left(\sum_{i=1}^{S_{\mathrm{obs}}}p_{i}^{2}\right)}^{1/1-2}$
Chao1 index	$S_{\mathrm{chao}1}=S_{\mathrm{obs}}+\frac{S_{n_{1}}(S_{n_{1}}-1)}{2(S_{n_{2}}+1)}$
ACE index ^1,2^	$S_{\mathrm{ACE}}=S_{\mathrm{abund}}+\frac{S_{\mathrm{rare}}}{C_{\mathrm{ACE}}}+\frac{S_{n_{1}}}{C_{\mathrm{ACE}}}\gamma_{\mathrm{ace}}^{2}$
Good’s coverage index	$C=1-\frac{S_{n_{1}}}{N}$
Margalef’s diversity index	$D_{\mathrm{Mg}}=\frac{S_{\mathrm{obs}}-1}{\ln(N)}$
Menhinick’s diversity index	$D_{\mathrm{Mn}}=\frac{S_{\mathrm{obs}}}{\sqrt{N})}$

^1^$C_{\mathrm{ACE}}=1-\frac{S_{n_{1}}}{N_{\mathrm{rare}}}$.

^2^$\gamma_{\mathrm{ace}}^{2}=\max\left[\frac{S_{\mathrm{rare}}}{C_{\mathrm{ACE}}}\frac{i(i-1)S_{n_{1}}}{\left(N_{\mathrm{rare}})(N_{\mathrm{rare}}-1\right)}-1,0\right]$.

**Fig. 3:**
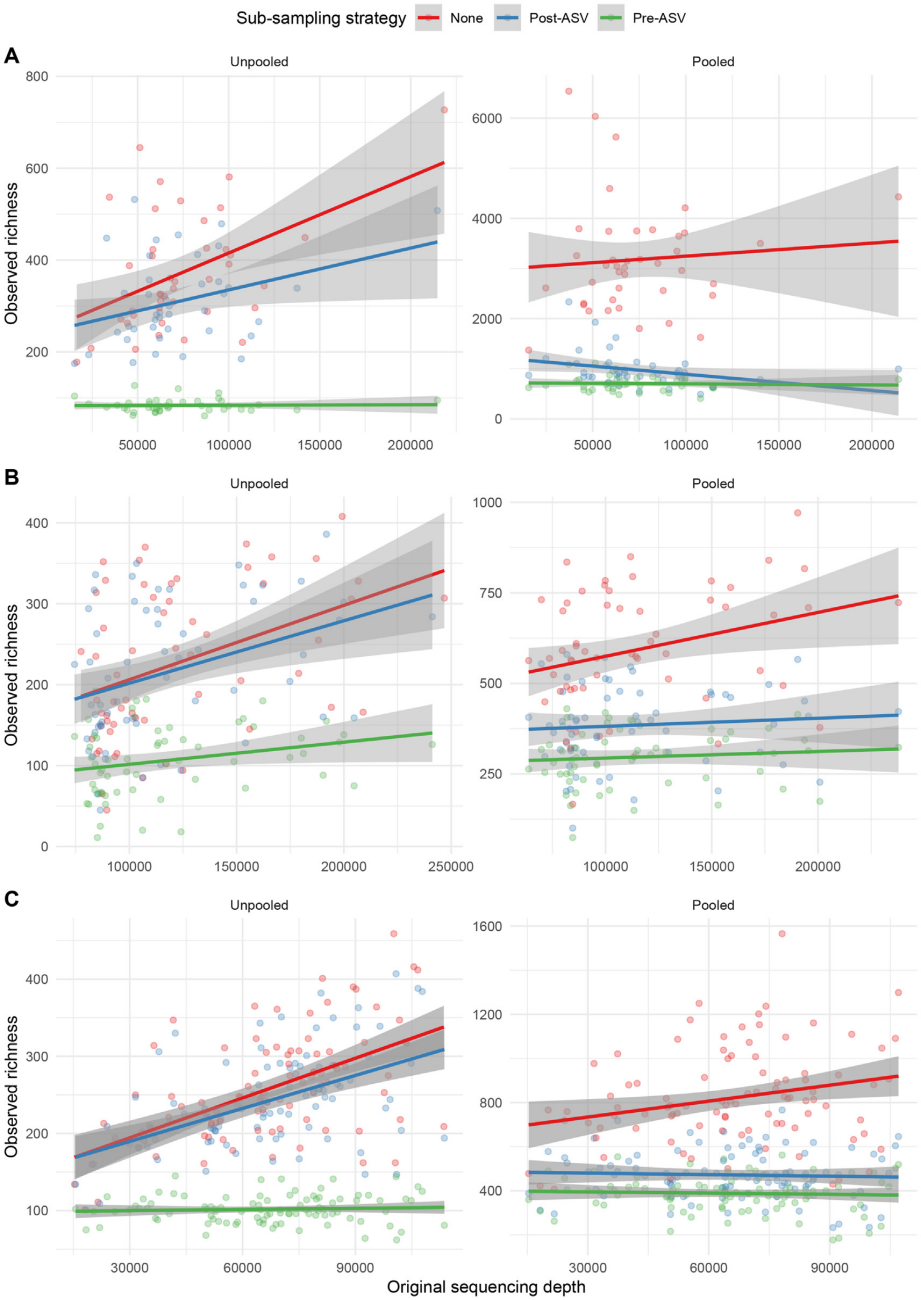
Scatter-plots with fitted regression line showing the association of observed richness with sequencing depth for data processed in a pooled and unpooled approach without prior for the data of **A** Almeida-Santos et al., 2021, **B** Manor et al., 2018 and **C** Aho et al., 2019.

**Fig. A.1:**
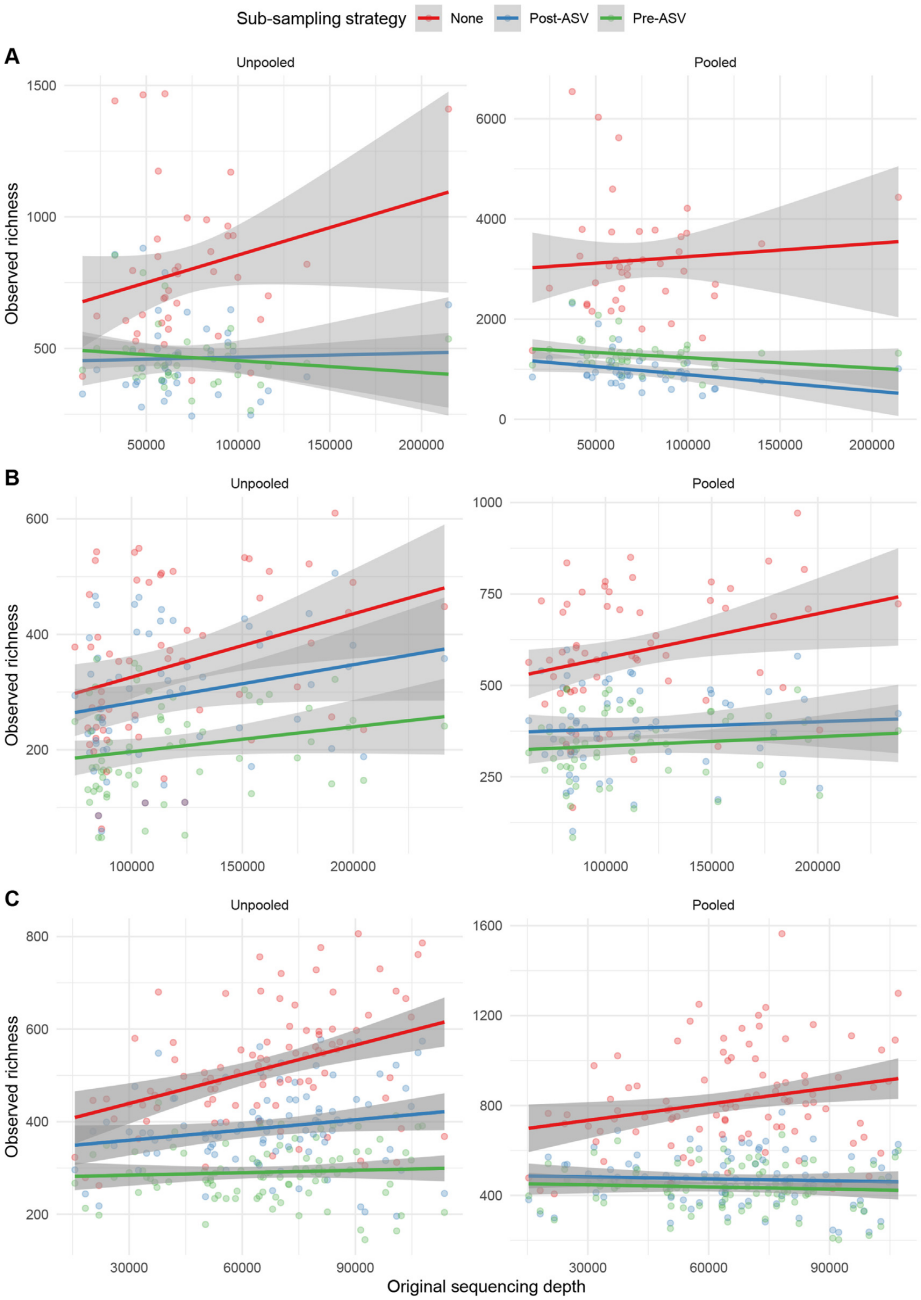
Scatter-plots with fitted regression line showing the association of observed richness with sequencing depth for data processed in a pooled and unpooled approach with prior for the data of **A** Almeida-Santos et al., 2021, **B** Manor et al., 2018 and **C** Aho et al., 2019.

We found that in the unpooled szenario without sub-sampling, observed richness was positively correlated with sequencing depth among all datasets. Post-ASV sub-sampling had almost no effect on observed richness as well as on the correlation with sequencing depth. Pre-ASV sub-sampling, however, substantially decreased observed richness in all three datasets. In addition, the correlation with the original sequencing depth almost vanished (Fig. 3 **C** and **A**) or was substantially reduced (Fig. 3 **B**). For the pooled scenario, we found that average richness was substantially higher as compared to the unpooled scenario when no sub-sampling was performed. As expected, this increase in richness was driven by a large increase in singleton reads (Table 3). A positive correlation with sequencing depth was still observed for two datasets, but not for dataset A. For the datasets C and B, the correlation with original sequencing depth decreased or vanished after sub-sampling, both pre- and post-ASV derivation. For dataset A, post-ASV sub-sampling resulted in a negative correlation (Table 3). The use of priors during processing produced similar results. In fact, the correlation between observed richness and sequencing depth was still observed. However, in contrast to scenarios without prior, post-ASV sub-sampling was able to reduce this correlation in unpooled scenarios (Fig. A.1 and Table A.1).

**Table 3:** Estimates of observed richness and correlation with original sequencing depth for data processed with DADA2. Estimates shown are calculated on data processed without use of a prior.

Study	Processing	Sub-sampling	ρ^1^	Mean observed richness (SD)	Mean singletons	
Aho et al., 2019 (**C**)	Unpooled	None	0.54	256.12 (70.30)	1.00	
		Post	0.50	241.65 (63.70)	17.99	
		Pre	0.07	101.80 (17.80)	0.43	
	Pooled	None	0.23	820.02 (217.00)	251.00	
		Post	−0.02	469.16 (110.00)	145.98	
		Pre	−0.01	388.27 (80.70)	125.14	
						
Almeida-Santos et al., 2021 (**A**)	Unpooled	None	0.34	372.90 (127)	10.80	
		Post	0.27	309.76 (94.7)	45.39	
		Pre	0.04	83.90 (14.1)	1.32	
	Pooled	None	0.08	3178.07 (1104)	2044.85	
		Post	−0.40	971.76 (352)	554.71	
		Pre	−0.01	704.66 (150)	407.93	
						
Manor et al., 2018 (**B**)	Unpooled	None	0.42	226.53 (92.20)	0.00	
		Post	0.36	216.05 (84.90)	12.33	
		Pre	0.27	106.53 (43.50)	0.00	
	Pooled	None	0.30	592.53 (166.00)	129.40	
		Post	0.09	384.10 (110.00)	94.82	
		Pre	0.15	296.28 (77.60)	66.93	

When data was processed with deblur, the correlation between observed richness and sequencing depth was observed as well. The strengths of correlation differed substantially between deblur and DADA2, with some correlations being stronger in the former and some in the latter (Table 3 and Table B.1). Sub-sampling, however, substantially reduced this correlation in all scenarios, regardless of whether it was performed pre- or post-ASV. Moreover, keeping singletons had little effect on the correlation between sequencing depth and observed richness.

The effects of pipeline settings in DADA2 were different for the various alpha diversity indices. While richness is directly associated to the observed number of ASVs, other alpha diversity indices may be less or more sensitive to changes in the number of ASVs. Chao1 estimates were almost identical to observed richness in unpooled scenarios. For pooled scenarios, the Chao1 estimates exceeded the estimates of observed richness as it was highly driven by the high number of singletons in the pooled data. Effects of sub-sampling, however, were similar to those for observed richness. As expected, Shannon diversity and Simpson diversity were less sensitive to differences between scenarios (pooled or unpooled) and sub-sampling strategy. The Good’s coverage estimator assumed almost full coverage of the sample in the unpooled scenario, irrespective of sub-sampling strategy. For the pooled scenario, the coverage in the data without sub-sampling was substantially lower and further decreased when sub-sampling was performed. Both Margalef’s and Menhinick’s diversity index showed increased diversity when using post-ASV sub-sampling, with a higher magnitude for the latter. Results in scenarios with prior were similar across all diversity measures. Most notably, across all indices, estimates after pre-ASV sub-sampling were higher as compared to scenarios without prior.

Across all indices , alpha diversity was substantially different when processed with deblur (as compared to DADA2), regardless of whether singletons were discarded or retained. DADA2 generally had lower diversity when processed without pooling. However, with pooling, the diversity increased and exceeded diversity obtained by deblur. Further, post-ASV and pre-ASV sub-sampling reduced diversity measures. Again, the effects where independent of keeping singletons.

**Fig. 4:**
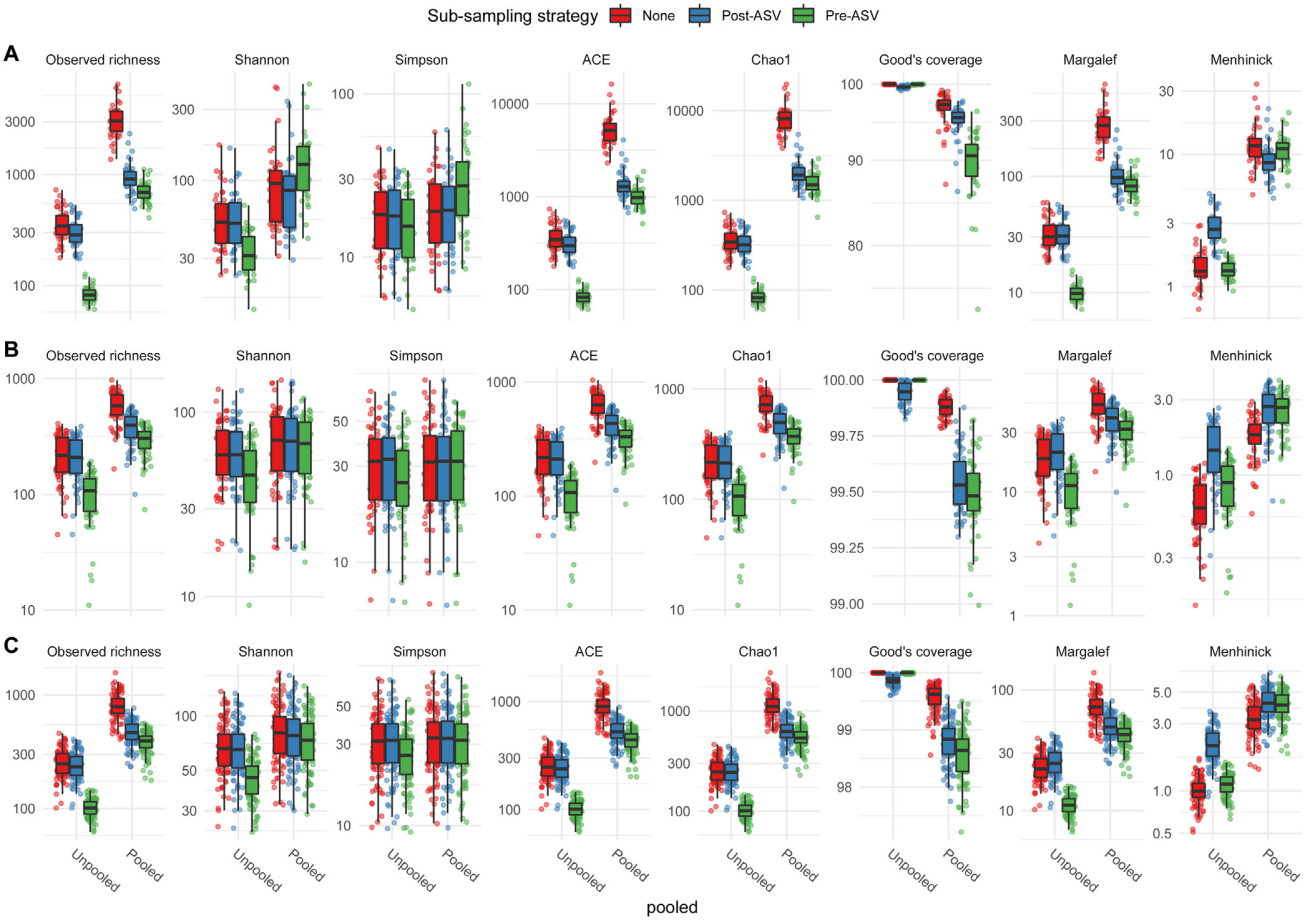
Distribution of alpha diversity indices for the full and saub-sampled data using DADA2 with pooled and unpooled processing without priors for the data of **A** Almeida-Santos et al., 2021, **B** Manor et al., 2018 and **C** Aho et al., 2019.

**Fig. 5:**
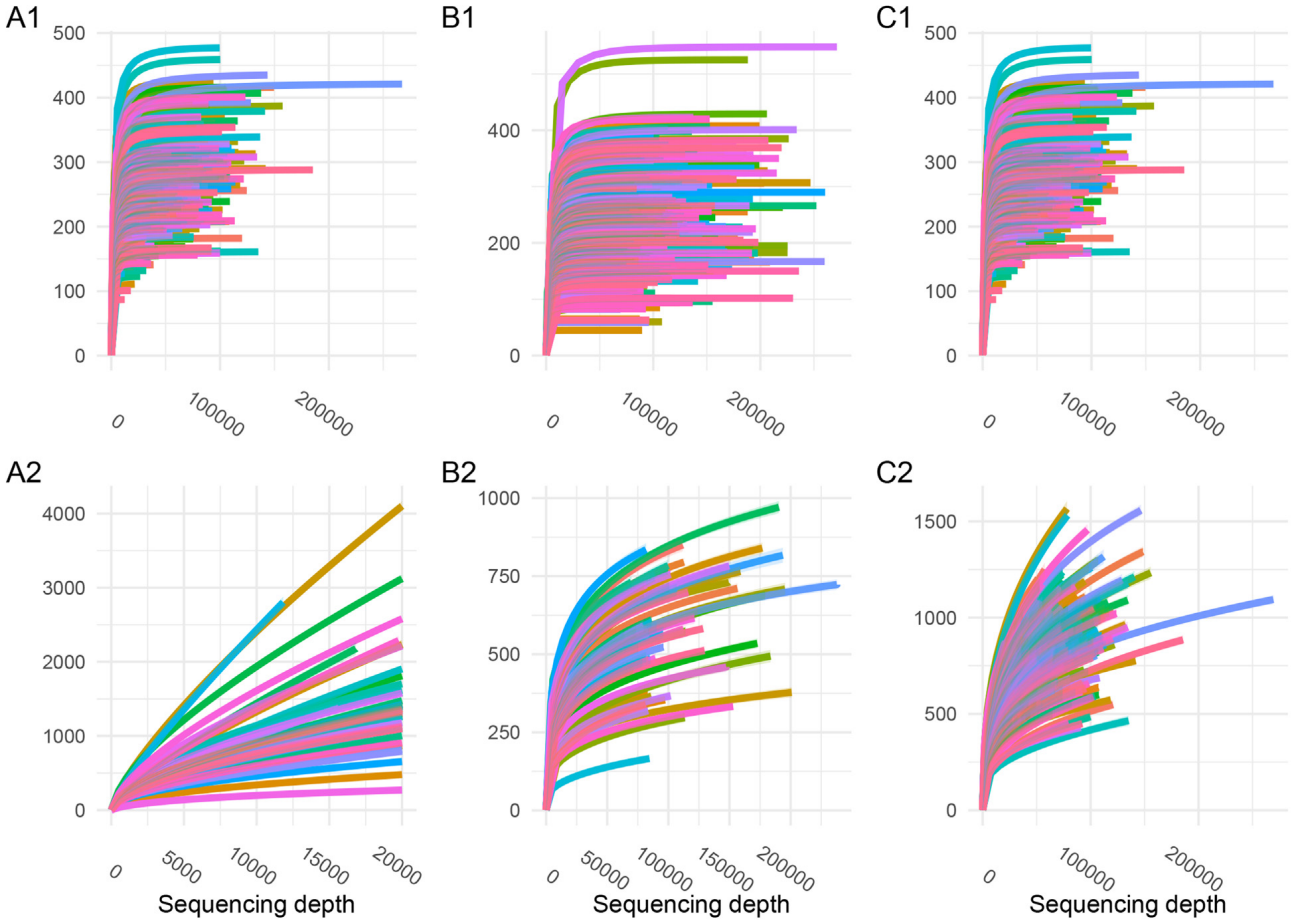
Rarefaction curves for the data of **A** Almeida-Santos et al., 2021, **B** Manor et al., 2018 and **C** Aho et al., 2019. processed either **1** unpooled or **2** pooled with DADA2.

Rarefaction curves showed considerable differences between scenarios. For all unpooled scenarios, the rarefaction curves showed a steep increase and an early plateau suggesting a sufficient coverage of the sample (Fig. 5 **A1**, **B1** and **C1**). This pattern was caused by the low (or not existent) number of singletons in data processed without pooling. However, when data were processed with pooling, the rarefaction curves changed across all datasets. In fact, none of the rarefaction curves seemed to be close to reaching a plateau (Fig. 5 **A2**, **B2** and **C2**), suggesting that the sample was not fully covered to detect all ASVs. These findings were in line with the conclusions drawn from Good’s coverage shown in Fig. 4.

**Fig. B.3:**
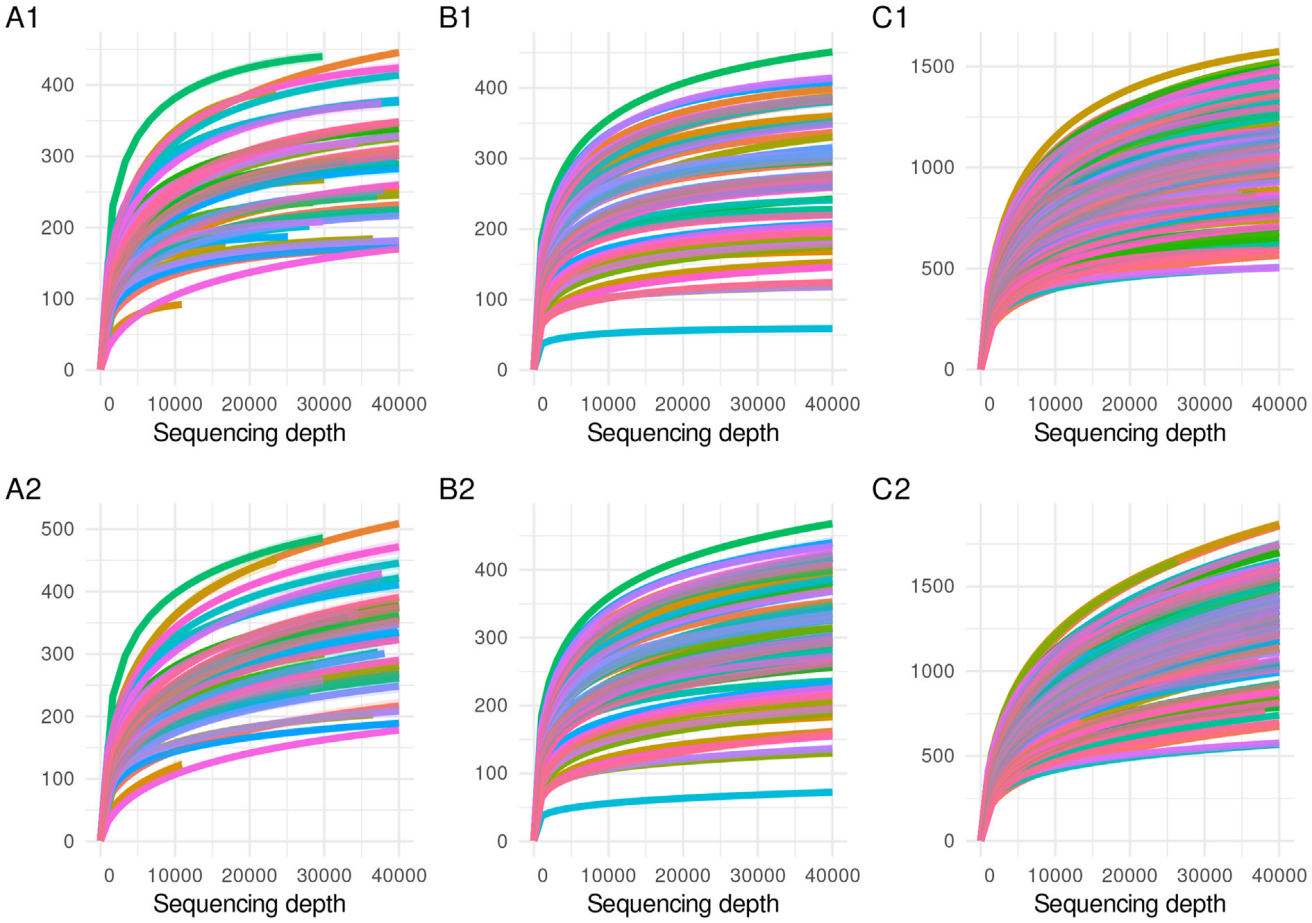
Rarefaction curves for the data of **A** Almeida-Santos et al., 2021, **B** Manor et al., 2018 and **C** Aho et al., 2019. processed either **1** without or **2** with keeping singletons with deblur.

When data was processed with deblur, rearefaction curves presented very different as compared to the rarefaction curves calculated on data produced by DADA2 (Fig. B.3). Instead of the early plateaus as observed in unpooled scenarios of DADA2, rarefaction curves showed smooth inclines with some curves showng tendencies to reach plateaus (e.g. Fig. B.3 C2).

## 4 Discussion

Our results show that pipeline setup affects both the richness estimates as well as the correlation of richness with the original sequencing depth. The observed effects are the interplay of two different processes.

First, denoising pipelines such as DADA2 aim to infer sample sequences with resolution up to differences of only a single nucleotide. Since it is not possible to distinguish whether a single read is a true sequence variant or a result of a sequencing error, the default DADA2 (and deblur) pipeline strictly treats singletons as sequencing errors and discards those reads. However, rarefaction is highly dependent on the number of singletons in a sample – the rarer the sequence, the more likely it is that it will be observed only once in a sample. When all singletons are discarded beforehand, effects of rarefaction are limited, as observed in the unpooled scenarios (Table 3). Furthermore, as richness estimation is highly dependent on singletons, richness estimates are not valid on those data. Although pre-ASV sub-sampling seems to have an effect on richness, these effects are the result of sequences that became singletons through sub-sampling (sub-sampling-induced singletons). While post-ASV sub-sampling kept those singletons, the pipeline discarded those sub-sampling-induced singletons in pre-ASV sub-sampling. Using the sequences obtained in the baseline dataset as prior information to inform the processing of the sub-sampled datasets prevented the pipeline from discarding sub-sampling-induced singletons – this approach minimized the differences in pre- and post-ASV sub-sampling.

The second process that affected the results is the independent sample-wise processing of the DADA2 pipeline. By default, samples are processed independently without sharing information across samples. This influences how singletons are handled during processing. The higher the sequencing depth, the higher the probability that a rare ASV is detected more than once. This in turn leads to an increased probability that a sequence is retained in a sample, while the same sequence is discarded in a sample with lower sequencing depth. This leads to a spurious correlation of sequencing depth with observed or estimated richness. Our analyses showed that this spurious correlation can be completely resolved by pooled processing of the data. When pooling the data, the information of sequences in one sample is used to inform the processing in all other samples. Therefore, a sequence that is observed once in one sample may still be retained when it is also present in at least one other sample. In fact, if the data are processed sample-wise, the individual sequencing depth is directly related to the probability of retaining rare ASVs in a sample. However, when pooling data, this direct relationship is broken. The probability of keeping rare sequences is now related to the overall sequencing depth of all samples and constant across samples. Using the prior information for sub-sampled datasets can also be seen as partial pooling of information between samples. Therefore, the results presented mainly focused on the comparison between pooled and unpooled data, as the mechanisms driving the effects are the same.

We show that pipeline settings substantially affect alpha diversity estimation. With the exception of the Shannon and the Simpson diversity index – both compound measures of richness and evenness – the magnitude of all diversity indices where highly influenced by the choice of pooled or unpooled processing. This influence was mainly driven by the number of singletons in the data. As all measures that aim to estimate richness incorporate a unique treatment of singleton (or very low abundant) reads (Table 2), these estimators are invalid for data processed with the standard unpooled pipeline. For example, the Chao1 estimator converges to the simple observed richness (Fig. 4) for unpooled data . Margalef’s and Menhinick’s diversity indices show even contradictory – and technically implausible – behaviour, with increased diversity after sub-sampling. This is caused by the decreasing number of overall reads in the denominator, while the number of observed ASVs stays almost constant.

We further present that the choice of either pooled or unpooled processing critically changed the pattern of rarefaction curves. It is a common strategy to use the sequencing depth at which these curves reach a plateau as sub-sampling depth. The curves in the unpooled data reach plateaus even at very low sequencing depth, which is an artifact of the treatment of singletons in the unpooled pipeline. For pooled data, none of the curves seem to be close to a plateau. Therefore, using rarefaction to decide on sequencing depth is not a valid strategy when using unpooled processing in DADA2.

Although deblur follows an approach similar to DADA2 and also utilizes independent sample-wise processing, not all results could be replicated in data processed by deblur. While the association of richness with sequencing depth was observed with similar magnitude, the correlation could be substantially reduced when sub-sampling was applied, independent if sub-sampling was performed before or after deriving ASVs. Furthermore, the magnitude of decrease in the correlation between sequencing depth and observed richness was higher when singletons were not discarded in the pipeline. The results suggest that, despite the similarity in approaches, DADA2 and deblur perform differently, with less severe effects for deblur.

Researchers using denoising pipelines who are interested in the quantification and investigation of alpha diversity, expecially richness, should be aware of these influences and adjust their pipelines or analyses accordingly. Our results further show that rarefaction or sub-sampling is not an adequate tool to ensure that richness is not related to sequencing depth, especially when using DADA2. Pooled processing in combination with sub-sampling, however, solves this issue by mitigating the spurious correlation induced by sample-wise processing. If one decides to make use of sub-sampling in data generated with DADA2, we argue, based on our conclusions, to use a pooled processing approach together with sub-sampling after derivation of ASVs to ensure highest sensitivity and data quality.

Although our analyses focused on DADA2 and deblur, the results extend to other pipelines that follow a similar denoising approach to call amplicon sequence variants. Several studies investigating biases and differences in bioinformatics pipelines showed that denoising pipelines had overall similar estimates of observed richness which were insensitive to most filtering steps [16], but differed significantly from classical approaches that construct operational taxonomic units [17].

We only used some filtering steps on overall read depth throughout our analyses. More strict filtering, e.g. the removal of ASVs below a specific count threshold, may affect the observed biases. Therefore, the analyses presented here should be used to investigate biases within studies, e.g. by rarefaction curves or investigation of correlation between diversity estimates and sequencing depth. Moreover, we argue that in situations where alpha diversity is subject to statistical analyses, sequencing depth should always be included as a covariate to adjust for confounding. When alpha diversity is only inspected exploratively, e.g. by visual inspection or descriptive statistics, researchers should also provide descriptive or visual information on possible systematic differences in sequencing depth between the subjects of interest.

At this point, it is crucial to note that the treatment of singletons in DADA2, deblur and other denoising pipelines is not an error or incorrect – in fact, it is possible to change this default behavior and keep singleton reads – but the aim of those pipelines is to infer sequence variants individually. On an individual level, it is almost impossible to infer whether an observed singleton is a valid singleton or a sequencing artifact and it may be reasonable to discard those sequences. Therefore, one may argue that denoising pipelines are not designed to estimate richness and should not be used for this purpose. Currently, it is still common to inspect richness in data obtained from denoising pipelines. Therefore, the results presented here are important for raising awareness of the described issues and enabling informed decisions about how to handle alpha diversity estimation for inference based on those estimates.

## Declaration of Competing Interest

The authors declare that they have no known competing financial interests or personal relationships that could have appeared to influence the work reported in this paper.

## Figures and Tables

**Fig. A.2:**
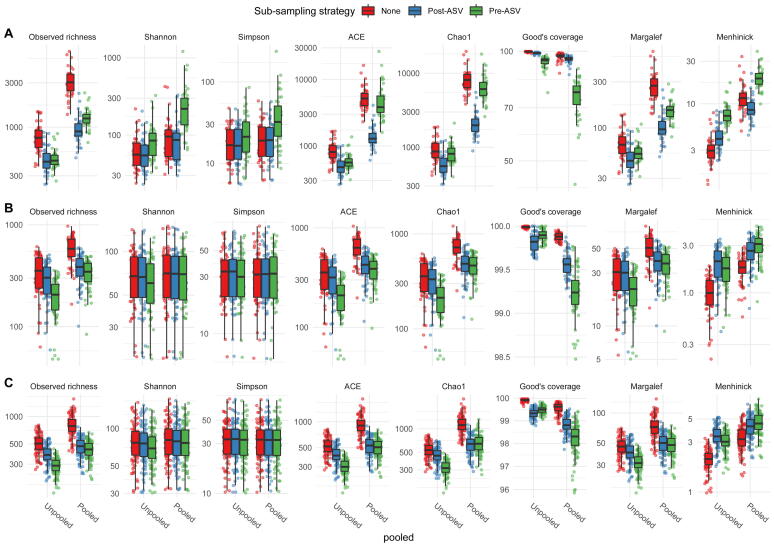
Distribution of alpha diversity indices for the original and rarefied data processed with deblur with and without keeping singleton reads for the data of **A** Almeida-Santos et al., 2021, **B** Manor et al., 2018 and **C** Aho et al., 2019.

**Table A.1:** Estimates of observed richness and correlation with original sequencing depth for data processed with DADA2 with priors.

Study	Processing	Sub-sampling	ρ^1^	Mean observed richness (SD)	Mean singletons	
Aho et al., 2019 (**C**)	Unpooled	None	0.22	796.98 (287.02)	190.44	
		Post	0.02	462.54 (152.98)	138.80	
		Pre	−0.09	466.27 (115.35)	228.85	
					
	Pooled	None	0.08	3178.44 (1104.34)	2045.37	
		Post	−0.40	975.1 (350.43)	560.27	
		Pre	−0.24	1283.32 (314.91)	1024.80	
						
Almeida-Santos et al., 2021 (**A**)	Unpooled	None	0.31	345.77 (136.82)	13.40	
		Post	0.23	293.47 (108.68)	39.62	
		Pre	0.24	204.55 (79.44)	15.23	
	Pooled	None	0.30	592.55 (166.24)	129.40	
		Post	0.10	383.4 (111.94)	94.47	
		Pre	0.15	337.98 (94.43)	95.28	
						
Manor et al., 2018 (**B**)	Unpooled	None	0.40	516.07 (123.08)	48.00	
		Post	0.22	386.8 (86.82)	84.05	
		Pre	0.13	290.88 (59.89)	46.23	
	Pooled	None	0.24	819.84 (216.97)	250.89	
		Post	−0.06	472.11 (109.51)	147.79	
		Pre	−0.05	436.06 (96.90)	158.07	

**Fig. B.1:**
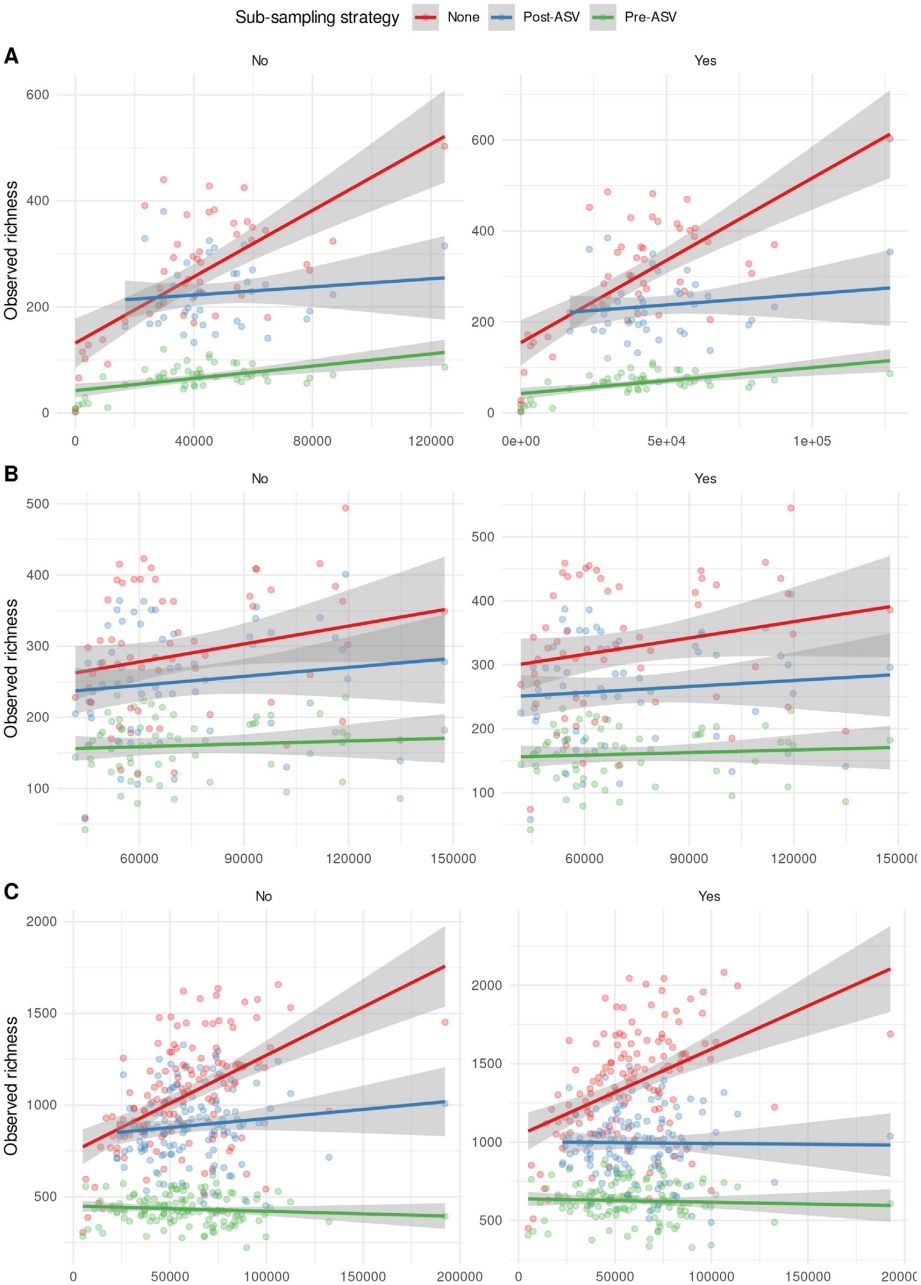
Scatter-plots with fitted regression line showing the association of observed richness with sequencing depth for data processed with deblur with and without keeping singleton reads for the data of **A** Almeida-Santos et al., 2021, **B** Manor et al., 2018 and **C** Aho et al., 2019.

**Fig. B.2:**
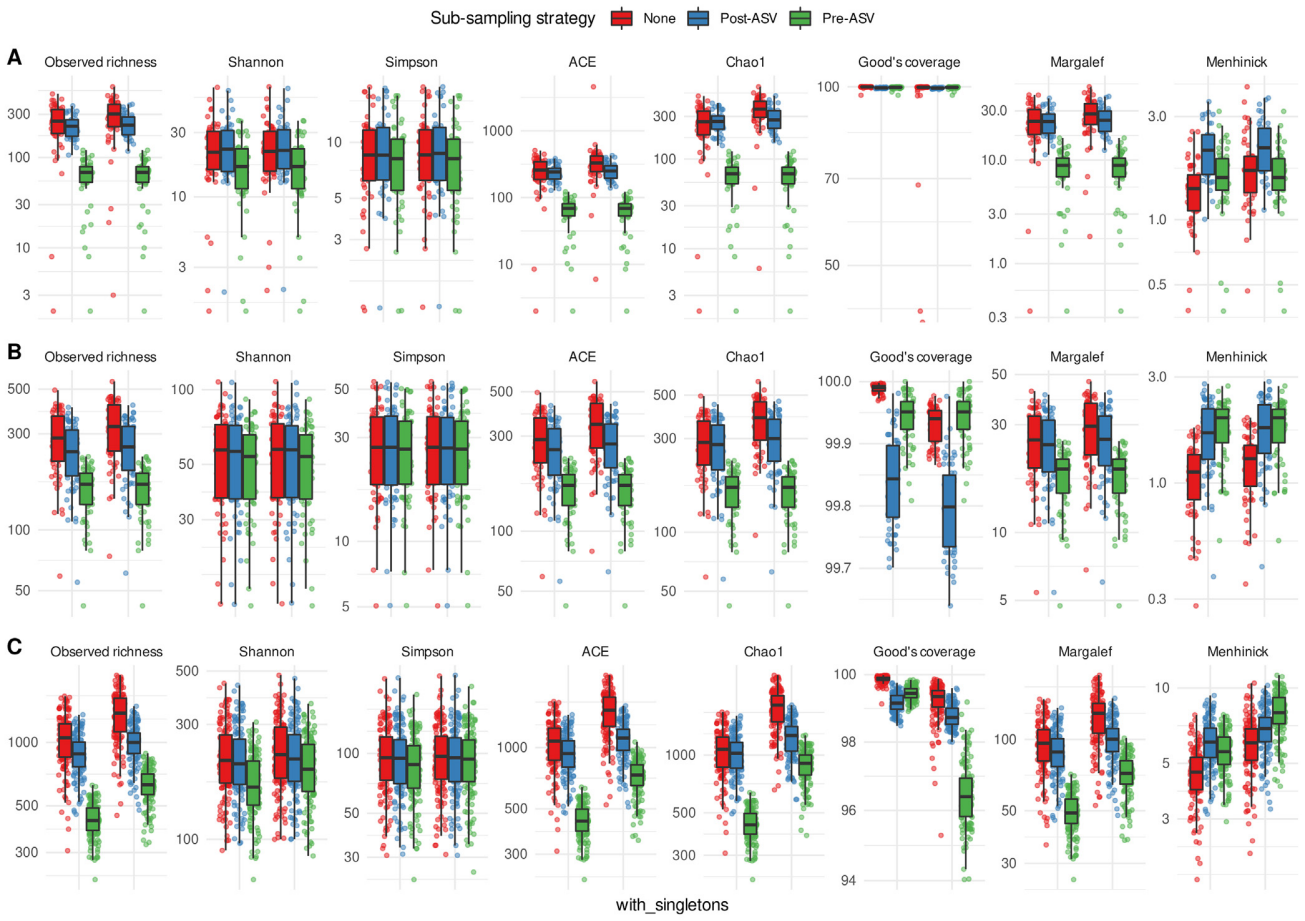
Distribution of alpha diversity indices for the full and sub-sampled data processed with deblur with and without keeping singleton reads for the data of **A** Almeida-Santos et al., 2021, **B** Manor et al., 2018 and **C** Aho et al., 2019.

**Table B.1:** Estimates of observed richness and correlation with original sequencing depth for data processed with deblur.

Study	Keep singletons	Sub-Sampling	ρ^1^	Mean observed richness (SD)	Mean singletons	
Aho et al., 2019 (**C**)	No	None	0.49	1050.16 (286.87)	61.35	
		Post	0.18	886.3 (193.87)	179.07	
		Pre	−0.12	433.32 (79.89)	33.55	
	Yes	None	0.44	1362.7 (345.77)	360.13	
		Post	0.03	993.16 (206.55)	262.38	
		Pre	−0.05	625.64 (118.78)	218.45	
						
Almeida-Santos et al., 2021 (**A**)	No	None	0.57	257.04 (110.88)	16.02	
		Post	0.05	221.05 (62.38)	49.12	
		Pre	0.35	64.51 (27.17)	3.84	
	Yes	None	0.60	297.18 (127.15)	60.24	
		Post	0.01	234 (66.45)	58.12	
		Pre	0.35	64.51 (27.17)	3.84	
						
Manor et al., 2018 (**B**)	No	None	0.21	288.48 (94.17)	7.62	
		Post	0.16	250.05 (79.62)	34.12	
		Pre	0.11	160.33 (42.45)	4.47	
	Yes	None	0.21	327.12 (100.4)	46.00	
		Post	0.11	260.78 (80.22)	42.75	
		Pre	0.11	160.33 (42.45)	4.47	

## Appendix A DADA2 results with prior

See Fig. A.1, Fig. A.2 and Table A.1.

## Appendix B Deblur results

See Fig. B.1, Fig. B.2, Fig. B.3 and Table B.1.
